# Improving communication with hard of hearing and D/deaf patients: a capacity-building intervention

**DOI:** 10.1093/eurpub/ckac129.178

**Published:** 2022-10-25

**Authors:** P Bodenmann, P Singy, M Graells, E Schmutz, T Sebaï, V Favre, J Richème-Roos, O Cantero, K Morisod, VS Grazioli

**Affiliations:** 1 Department of Vulnerabilities and Social Medicine, Center for Primary Care and Public Health, University of Lausanne, Lausanne, Switzerland; 2 Psychiatric Liason Service, Lausanne University Hospital, Lausanne, Switzerland

## Abstract

**Background:**

D/deaf and hard of hearing (D&HoH) populations are disproportionally affected by physical and mental health problems while facing barriers to accessing health services. These barriers stem from communication challenges with healthcare providers, who are often unprepared to meet their specific needs. This study aimed to develop and evaluate an intervention to improve healthcare providers’ skills to communicate with these patients.

**Methods:**

This study featured a participative action research design. Consistently, the intervention was developed through iterative phases together with the target populations and key stakeholders. The finale version was tested in healthcare workers in Canton of Vaud in Switzerland. Participants completed a questionnaire before (T0) and 6 months after (T1) the intervention, assessing perceived knowledge of deafness and hard of hearing and tools to improve communication, self-efficacy on how to communicate with D&HoH patients and institutional benefits (application frequency of communication rules and tools).

**Results:**

The final intervention aimed to increase participants’ 1) awareness of D&HoH experience and communication needs, 2) knowledge of the tools and basic rules to improve communication. Two D&HoH trainers led one half-day intervention among 28 healthcare providers (e.g., nurses, pharmacists; mean age=43.6). Paired-sample t-tests revealed significant increases in knowledge between T0 and T1, t (23) = -7.81, p < .001 and in self-efficacy, t (24) = -10.23, p < .001, whereas there was no significant difference between institutional benefits at T0 and T1.

**Conclusions:**

Although findings suggest the intervention is a promising means to increase perceived knowledge and self-efficacy on how communicating with D&HoH patients, complementary approaches, such as a resource person within the institutions providing day-to-day support to the teams besides the intervention, may be necessary to induce institutional changes.

**Key messages:**

• Future research should implement the intervention more broadly within inpatient and outpatient settings in Switzerland to increase knowledge on how communicating with D&HoH patients.

• Intervention implementation should be complemented by an additional structural approach to induce sustainable changes in practice and evaluated over 12 months to ensure sustainability.

